# Coronary artery distensibility is impaired in HIV patients without significant coronary atherosclerosis

**DOI:** 10.1186/1532-429X-18-S1-O53

**Published:** 2016-01-27

**Authors:** Lia Petrose, Micaela Iantorno, Sahar Soleimanifard, Michael Schär, Richard Moore, Gary Gerstenblith, Robert Weiss, Allison Hays

**Affiliations:** 1 Medicine, Johns Hopkins, Clarksville, MD USA; 2 Radiology, Johns Hopkins, Baltimore, MD USA

## Background

HIV infection is associated with an increased likelihood of coronary artery disease (CAD) and related events, but the underlying mechanisms are not well understood. Reduced coronary distensibility (CD) may contribute to the development of atherosclerosis and is present in CAD patients^1^. While aortic distensibility (AD) is known to be reduced in HIV^2^, CD in HIV patients has not been studied. We tested the hypothesis that CD is reduced in HIV patients, even before the development of CAD, and that the reduction in CD correlates with changes in AD, reflecting diffuse changes in vascular material properties in HIV+ patients.

## Methods

20 healthy adults and 13 HIV+ patients with a zero coronary artery calcium score on CT underwent 3T MRI to measure CD and AD, as previously described to evaluate the relationship between CD and AD. A subgroup of age-matched healthy subjects (N = 13, age = 46.2 ± 2.6 yrs, mean+/-SEM), were compared to the 13 HIV+ patients (from above, age 51.8 ± 1.8 yrs, p = 0.1 vs healthy) to compare CD and AD between groups. A proximal or mid segment of a major coronary artery and a cross section of the ascending aorta were imaged using cine spiral MRI for area measurements. Images were analyzed for cross-sectional area during systole and diastole using semi-automated software (Cine vs3.15.17, General Electric), and distensibility(mmHg^-1^) was determined: [(systolic lumen area-diastolic lumen area)]/(pulse pressure multiplied by diastolic lumen area)^3^.

## Results

**Figure 1:**
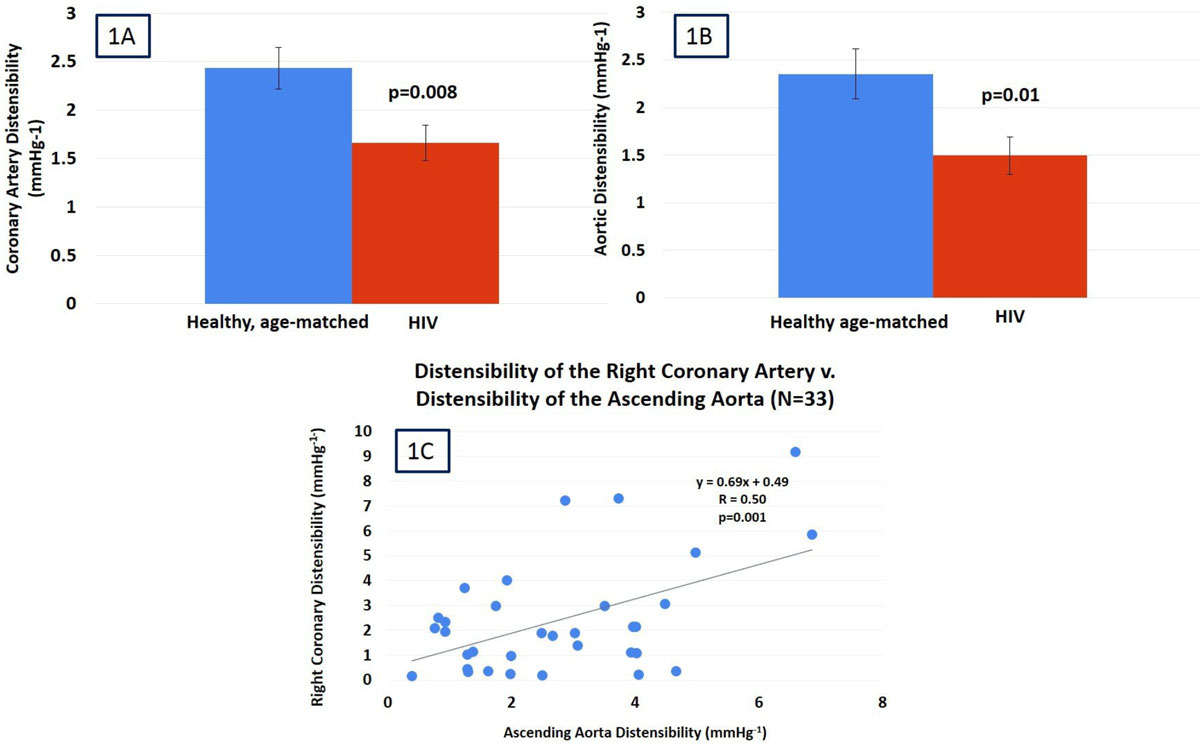
Figure 1

CD was reduced in HIV+ patients, (1.66 ± 0.17 mmHg^-1^) as compared to that in healthy subjects (2.43 ± 0.21 mmHg^-1^, p = 0.008, Fig 1A). Similarly, AD was decreased in HIV+ patients, (1.49 ± 0.20 mmHg^-1^) as compared to that in healthy subjects (2.35 ± 0.23 mmHg^-1^, p = 0.01, Fig 1B). There was no significant difference in pulse pressure between the two groups. There was a significant positive relationship between AD and CD (R = 0.50, p = 0.001, Figure 1C).

## Conclusions

Coronary distensibility is reduced in HIV+ patients even before the development of detectable CAD and is similar in extent to the CD reduction reported in HIV- patients with known CAD^1^. We confirm prior findings that AD is reduced in HIV (with 0 Calcium score) and demonstrate for the first time a significant positive relationship between AD and CD. The findings suggest that 1) vascular material properties are altered systemically in HIV+ patients even before CAD development and 2) a common mechanism of subclinical disease exists between the two vascular beds in HIV, possibly contributing to the increased risk for the development of atherosclerosis and cardiac events in HIV+ patients.

